# 

**Published:** 1892-06

**Authors:** 


### LITERARY.

### HARMONIZED MELODIES.

Budget for April, published monthly, by F. Trifet, 408 Washington Street, Boston.

We are under obligations to the publisher for the above collection of more than 400 songs and ballads, embracing the "newest of the new and the choicest of the old," arranged with "all the parts." Sent by mail anywhere in the United States, postpaid, for 60 cents.

The wonder is how does the enterprising publisher manage to do it ?

The authors of the following instructive essays will accept our thanks for copies.

MEDICAL EDUCATION AND LEGISLATION. By Prof. George J. Engleman, M.D., St. Louis.

EUROPHEN, A NEW IODINE COMPOUND, ANTISEPTIC ANTISPHILITIC AND SUBSTITUTE FOR IODOFORM. By Dr. F. Goldmann.

THIRTY-TWO UNSELECTED ABDOMINAL SECTIONS. By Thomas Opie, M.D., Baltimore, Md.

BRIGHT'S DISEASE. By L. Boemer, St. Louis.

